# The complete mitochondrial genome of *Tenomerga trabecula* (Coleoptera: Archostemata: Cupedidae) and phylogenetic analysis among Coleoptera

**DOI:** 10.1080/23802359.2021.2025162

**Published:** 2022-02-06

**Authors:** Lijie Jin, Yujie Li, Ming Bai

**Affiliations:** a Key Laboratory of Zoological Systematics and Evolution, Institute of Zoology, Chinese Academy of Sciences, Beijing, China; b Jiangsu Key Laboratory for Biodiversity and Biotechnology, College of Life Sciences, Nanjing Normal University, Nanjing, China

**Keywords:** Cupedidae, mitogenome, phylogeny, *Tenomerga trabecula*

## Abstract

*Tenomerga trabecula* belongs to the Cupedidae family of Coleoptera. The first complete mitogenome of Cupedidae is reported in this paper. The genome is 16,741 bp in length and contains the typical 37 genes with 22 transfer RNA genes, 13 protein-coding genes, and 2 ribosomal RNA genes, which are arranged in the same order as the putative ancestor of beetles. The average base composition of the mitogenome is 42.9% for A, 14.5% for C, 8.7% for G, and 33.9% for T. The percentage of A + T is 76.8%. The genome organization, nucleotide composition, and codon usage are similar to other beetles. Phylogenetic analysis shows that Archostemata is monophyletic. Myxophaga, Adephaga, and Polyphaga are also monophyletic.

*Tenomerga trabecula* Neboiss, 1984 (Coleoptera: Archostemata: Cupedidae), is distributed in Taiwan, Fujian, and Hong Kong, China (Neboiss 1984; Rodriguez-Miron and Lopez-Perez 2019). Within 45 extant species of Archostemata in the world, Cupedidae is the richest family with 36 species (Rodriguez-Miron and Lopez-Perez 2019). Only one complete mitogenome from Archostemata (Ommatidae: *Tetraphalerus bruchi*, EU877953) has been sequenced to date (Sheffield et al. 2008).

The specimen used in this study was collected using a flight interception trap (FIT) in Hong Kong (22.389327°N, 114.081250°E) and deposited in the National Zoological Museum of China, Institute of Zoology, Chinese Academy of Sciences (IOZ, CAS) (baim@ioz.ac.cn), with voucher number IOZ(E)2081544. It was identified by Yandong Chen of IOZ, CAS. Genomic DNA was extracted by a DNeasy Blood and Tissue kit (Qiagen, Germany) and deposited in a refrigerator at −20 °C at IOZ, CAS.

The complete mitogenome of *T. trabecula* was sequenced by the Illumina HiSeq 6000 platform with 400 bp insert size and a pair-end 150 bp sequencing strategy. The sequence reads were first filtered by the programs following Bolger et al. (2014); then, the remaining high-quality reads were assembled using mitoZ (Meng et al. 2019) and Getorganelle (Dierckxsens et al. 2017). The annotation of genes was performed by Geneious 8.0.5 software (Kearse et al., 2012) and MITOS (Bernt et al. 2013).

The mitogenome of *T. trabecula* was found to be a double-stranded circular molecule with 16,741 bp in length (GenBank accession number MW820160), containing the entire set of 37 genes usually present in most insect mtDNA (13 PCGs, 22 tRNA genes, and 2 rRNA genes) and a large non-coding region (control region). Twenty-three genes were transcribed on the majority strand (J-strand), whereas the others were oriented on the minority strand (N-strand). The overall organization of the mitogenome of *T. trabecula* is very compact, and numerous overlaps between genes are found. Five PCGs (ND2, ATP6, ND4, ND6, ND1) begin with an ATA start codon, and 4 PCGs (ATP8, ND3, ND5, ND4L) begin with an ATT start codon, while COX2, COX3, and CYTB begin with an ATG start codon. Nine PCGs terminate with an TAA stop codon, and only ND1 terminates with an TAG stop codon, whereas three genes (ATP6, ND6, COX2) terminate with incomplete T stop codons, which is frequently found in the mitogenome of beetles (Sheffield et al. 2008).

The aligned data from each locus were concatenated with PhyloSuite v1.2.2 (Zhang et al. 2020). A phylogenetic IQ-tree was constructed using PhyloSuite under the TVM + F + I + G4 model with 1000 bootstrap replicates (Nguyen et al. 2015). Nodal support values were estimated using the SH-aLRT branch test, which is computationally efficient and relatively unbiased.

The IQ-tree based on the concatenated 13 PCGs (Figure 1) shows that Coleoptera were recovered as monophyletic. The available suborder rank of Coleoptera ((Myxophaga + Adephaga) + (Archostemata + Polyphaga)) was resolved with strong support. This topology supports the monophyly of four suborders and the sister group relationships between Adephaga and Myxophaga and between Polyphaga and Archostemata, which is consistent with the analysis using the mitogenomes of 50 representative species from Coleoptera (Zhang et al. 2016).

**Figure 1.**
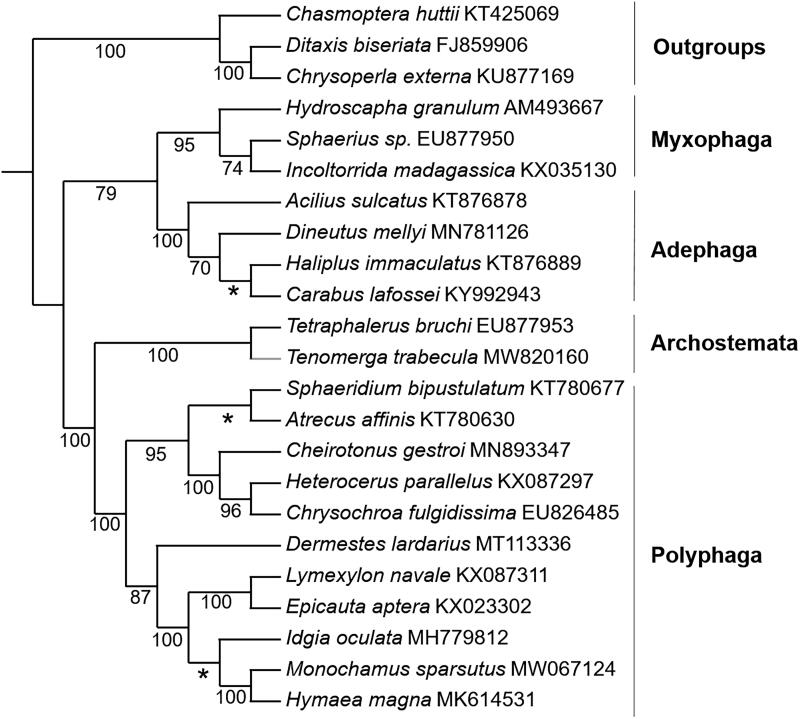
The IQ-tree based on 13PCGs combined data sets. Numbers on nodes indicate bootstraps. Red branch is the new data in this study.

## Data Availability

The genome sequence data supporting the findings of this study are openly available in the NCBI GenBank at https://www.ncbi.nlm.nih.gov/ under accession number MW820160. The associated BioProject, SRA, and Bio-Sample numbers are PRJNA750629, SRR15293804, and SAMN20448115, respectively.

## References

[CIT0001] Bernt M, Donath A, Jühling F, Externbrink F, Florentz C, Fritzsch G, Pütz J, Middendorf M, Stadler PF. 2013. MITOS: improved de novo metazoan mitochondrial genome annotation. Mol Phylogenet Evol. 69(2):313–319. 22982435 10.1016/j.ympev.2012.08.023

[CIT0002] Bolger AM, Lohse M, Usadel B. 2014. Trimmomatic: a flexible trimmer for Illumina sequence data. Bioinformatics. 30(15):2114–2120. 24695404 10.1093/bioinformatics/btu170 PMC4103590

[CIT0003] Dierckxsens N, Mardulyn P, Smits G. 2017. NOVOPlasty: de novo assembly of organelle genomes from whole genome data. Nucleic Acids Res. 45(4):e18. 28204566 10.1093/nar/gkw955 PMC5389512

[CIT5476486] Kearse M, Moir R, Wilson A, Stones-Havas S, Cheung M, Sturrock S, Buxton S, Cooper A, Markowitz S, Duran C, et al. 2012. Geneious Basic: an integrated and extendable desktop software platform for the organization and analysis of sequence data. Bioinformatics. 28(12):1647–1649. doi:10.1093/bioinformatics/bts199. 22543367 22543367 PMC3371832

[CIT0004] Meng G, Li Y, Yang C, Liu S. 2019. MitoZ: a toolkit for animal mitochondrial genome assembly, annotation and visualization. Nucleic Acids Res. 47(11):e63. 30864657 10.1093/nar/gkz173 PMC6582343

[CIT0005] Neboiss A. 1984. Reclassification of *Cupes* Fabricius (S Lat), with descriptions of new genera and species (Cupedidae, Coleoptera). System Entomol. 9(4):443–477.

[CIT0006] Nguyen LT, Schmidt HA, von Haeseler A, Minh BQ. 2015. IQ-TREE: a fast and effective stochastic algorithm for estimating maximum-likelihood phylogenies. Mol Biol Evol. 32(1):268–274. 25371430 10.1093/molbev/msu300 PMC4271533

[CIT0007] Rodriguez-Miron GM, Lopez-Perez S. 2019. A new reticulated beetle (Coleoptera: Cupedidae) from Mexico with a catalogue of Cupedidae species of the world. Zootaxa. 1:4567. 10.11646/zootaxa.4567.1.8 31716443

[CIT0008] Sheffield NC, Song H, Cameron SL, Whiting MF. 2008. A comparative analysis of mitochondrial genomes in Coleoptera (Arthropoda: Insecta) and genome descriptions of six new beetles. Mol Biol Evol. 25(11):2499–2509. 18779259 10.1093/molbev/msn198 PMC2568038

[CIT0009] Zhang D, Gao F, Jakovlic I, Zou H, Zhang J, Li WX, Wang GT. 2020. PhyloSuite: An integrated and scalable desktop platform for streamlined molecular sequence data management and evolutionary phylogenetics studies. Mol Ecol Resour. 20(1):348–355. 31599058 10.1111/1755-0998.13096

[CIT0010] Zhang H, Liu N, Han Z, Liu J. 2016. Phylogenetic analyses and evolutionary timescale of Coleoptera based on mitochondrial sequence. Biochem Syst Ecol. 66:229–238.

